# Automated Multi-View Multi-Modal Assessment of COVID-19 Patients Using Reciprocal Attention and Biomedical Transform

**DOI:** 10.3389/fpubh.2022.886958

**Published:** 2022-05-25

**Authors:** Yanhan Li, Hongyun Zhao, Tian Gan, Yang Liu, Lian Zou, Ting Xu, Xuan Chen, Cien Fan, Meng Wu

**Affiliations:** ^1^Electronic Information School, Wuhan University, Wuhan, China; ^2^Department of Gastroenterology, The Second Affiliated Hospital of Chongqing Medical University, Chongqing, China; ^3^Chongqing Key Laboratory of Ultrasound Molecular Imaging, The Second Affiliated Hospital of Chongqing Medical University, Chongqing, China; ^4^Department of Ultrasound, Zhongnan Hospital of Wuhan University, Wuhan, China; ^5^School of Economics and Management, Wuhan University, Wuhan, China; ^6^Beijing Genomics Institute (BGI) Research, Shenzhen, China

**Keywords:** COVID-19, deep learning, multi-view, multi-modal, computer aided diagnosis

## Abstract

Automated severity assessment of coronavirus disease 2019 (COVID-19) patients can help rationally allocate medical resources and improve patients' survival rates. The existing methods conduct severity assessment tasks mainly on a unitary modal and single view, which is appropriate to exclude potential interactive information. To tackle the problem, in this paper, we propose a multi-view multi-modal model to automatically assess the severity of COVID-19 patients based on deep learning. The proposed model receives multi-view ultrasound images and biomedical indices of patients and generates comprehensive features for assessment tasks. Also, we propose a reciprocal attention module to acquire the underlying interactions between multi-view ultrasound data. Moreover, we propose biomedical transform module to integrate biomedical data with ultrasound data to produce multi-modal features. The proposed model is trained and tested on compound datasets, and it yields 92.75% for accuracy and 80.95% for recall, which is the best performance compared to other state-of-the-art methods. Further ablation experiments and discussions conformably indicate the feasibility and advancement of the proposed model.

## 1. Introduction

In December 2019, coronavirus disease 2019 (COVID-19) broke out and began spreading to many countries around the globe, causing the ongoing coronavirus pandemic (1, 2). During the COVID-19 pandemic, lung imaging has played a crucial role in clinical care and epidemiological studies. COVID-19 has a significant impact on patients' respiratory systems, causing changes in the parenchyma of the lungs. Therefore, lung imaging is not only an effective technique for detecting COVID-19 but can also provide important information for clinicians to judge the severity of COVID-19 in patients through imaging features (3, 4). Common medical imaging techniques include computed tomography (CT), X-ray, and ultrasound. Among those medical imaging modalities, ultrasound possesses many advantages over others, including a high equipment penetration depth, ease of operation, the absence of radiation exposure, portability, the ability to perform real-time diagnosis, affordability, etc. (5–8). It is widely used as an additional screening and practical imaging method.

However, utilizing lung ultrasound images to assess the severity of COVID-19 patients is a complex and time-consuming task. Besides, manual judgments of sonographers will be influenced by inevitable subjective factors, leading to the omittance of inapparent image features. Deep learning (9) has achieved great success in the medical imaging domain (10–12). Deep neural network (DNN) models (9) even outperform human experts in the diagnosis of some diseases (13–15). Recently, many researchers have applied deep learning to the diagnosis or evaluation of COVID-19 (16–20). Wang et al. (21) proposed the COVID-Net to classify X-ray images into normal, pneumonia, and COVID-19. Chen et al. (22) established a deep-learning-based diagnostic system to identify COVID-19 pneumonia. Aboutalebi et al. (23) leveraged transfer learning to transfer representational knowledge for predicting the airspace severity of a SARS-CoV-2 positive patient based on CXR images. Amyar et al. (24) proposed a new multitask deep learning model to jointly identify COVID-19 patients and segment COVID-19 lesions from chest CT images. Park et al. (25) proposed a novel multi-task vision transformer that leverages low-level CXR feature corpus for COVID-19 diagnosis and severity quantification. Sharifrazi et al. (26) proposed a model fusing convolutional neural network (CNN), support vector machine (SVM), and Sobel filter to detect COVID-19 using X-ray images. Ayoobi et al. (27) proposed to predict new cases and death rates of COVID-19 patients in different time spans utilizing multiple deep learning methods. Asgharnezhad et al. (28) proposed to quantify the competency of DNNs for generating reliable uncertainty estimates for COVID-19 diagnosis by introducing novel performance metrics. Alizadehsani et al. (29) proposed to cope with insufficient labeled COVID-19 data by introducing a semi-supervised classification method relying on Sobel edge detection and generative adversarial networks (GANs). Similarly, to mitigate the shortage of medical resources, Joloudari et al. (30) proposed DNN-GFE which combined DNNs with a Global Feature Extractor (GFE) for COVID-19 diagnosis. Khozeimeh et al. (31) proposed to deal with unbalanced data by introducing a data augmentation procedure based on autoencoders (AEs) and constructing CNN-AE to automatically diagnose COVID-19 cases. However, there are still several flaws in existing methods. (1) Most studies (19, 21, 23, 24) focus on chest X-ray and CT imaging, and little work takes lung ultrasound images into consideration. (2) Existing methods (17, 19, 21, 23, 24, 32) mostly leverage single-view images as input while it is more rational to exploit multi-view ones. (3) Existing methods (17, 19, 21, 23–25, 32) mostly utilize data of single modality (unitary CT, X-ray, ultrasound, or other modalities) while multi-modal data are conductive to offer more information.

To mitigate the aforementioned flaws, in this paper, we propose an automated multi-view multi-modal model to analyze the severity in COVID-19 patients. The proposed model receives dual-view ultrasound image pairs and biomedical indices of patients to automatically conduct the comprehensive severity assessment tasks. We also propose reciprocal attention module and biomedical transform module, respectively, to extract and integrate multi-view and multi-modal features.

Briefly, the main contributions of our model are as follows:

1. A novel multi-view multi-modal DNN is proposed. To the best of our knowledge, we are one of the forerunners to use both multi-view and multi-modal model for severity assessment in COVID-19 patients. Our proposed model has been evaluated on this dataset and outperformed all other state-of-the-art methods.
2. A novel reciprocal attention module is proposed. Reciprocal attention module embedded with attention mechanism (33) rationally explores the inherent connection between ultrasound images of multiple views, generating attention features.
3. A novel biomedical transform module is proposed. The biomedical transform module incorporates information of biomedical indices into ultrasound features, producing comprehensive hybrid features for assessment.

## 2. Materials and Methods

### 2.1. Data Acquisition

We collected data from a total of 164 patients, ranging in age from 17 to 87 years old, with 48.17% males and 51.83% females from Zhongnan Hospital of Wuhan University and Leishenshan Hospital to form the two datasets of COVID-19 patients. We collected 1,712 ultrasound images from the patients and simultaneously, we collected the corresponding biochemical indices related to pneumonia, including lymphocytes, c-reactive protein, lactate dehydrogenase, procalcitonin, and interleukin 6. See Table 1 for detailed information.

**Table 1:** The clinical information related to coronavirus disease 2019 (COVID-19) patients.

**Characteristics**	**Total**
Age, mean (s.d.) (years)	58.67(12.07)
Gender, *n* (%)	
Male	79 (48.17)
Female	85 (51.83)
Patient's classification, n (%)	
Mild	125 (76.22)
Severe	39 (31.2)
Laboratory test (the latest laboratory test in lung ultrasound examination)	
LYM^*a*^, median (range, Q1-Q3) (10^9^/L)	1.6 (0.28, 3.68)
CRP^*b*^, median (range, Q1-Q3) (mg/L)	2.06 (0.5, 278.11)
LDH^*c*^, median (range, Q1-Q3) (IU/L)	193 (116, 847)
PCT^*d*^, median (range, Q1-Q3) (ng/mL)	0.04 (0.01, 9.25)
IL-6^*e*^, median (range, Q1-Q3) (pg/mL)	1.71 (1.5, 1716)

a *LYM, lymphocytes*.

b *CRP, C-reactive protein*.

c *LDH, lactate dehydrogenase*.

d *PCT, procalcitonin*.

e *IL-6, interleukin 6*.

*Continuous variables that conform to the normal distribution are described as the mean (s.d.), otherwise, using the median and interquartile range values*.

**Figure 1:**
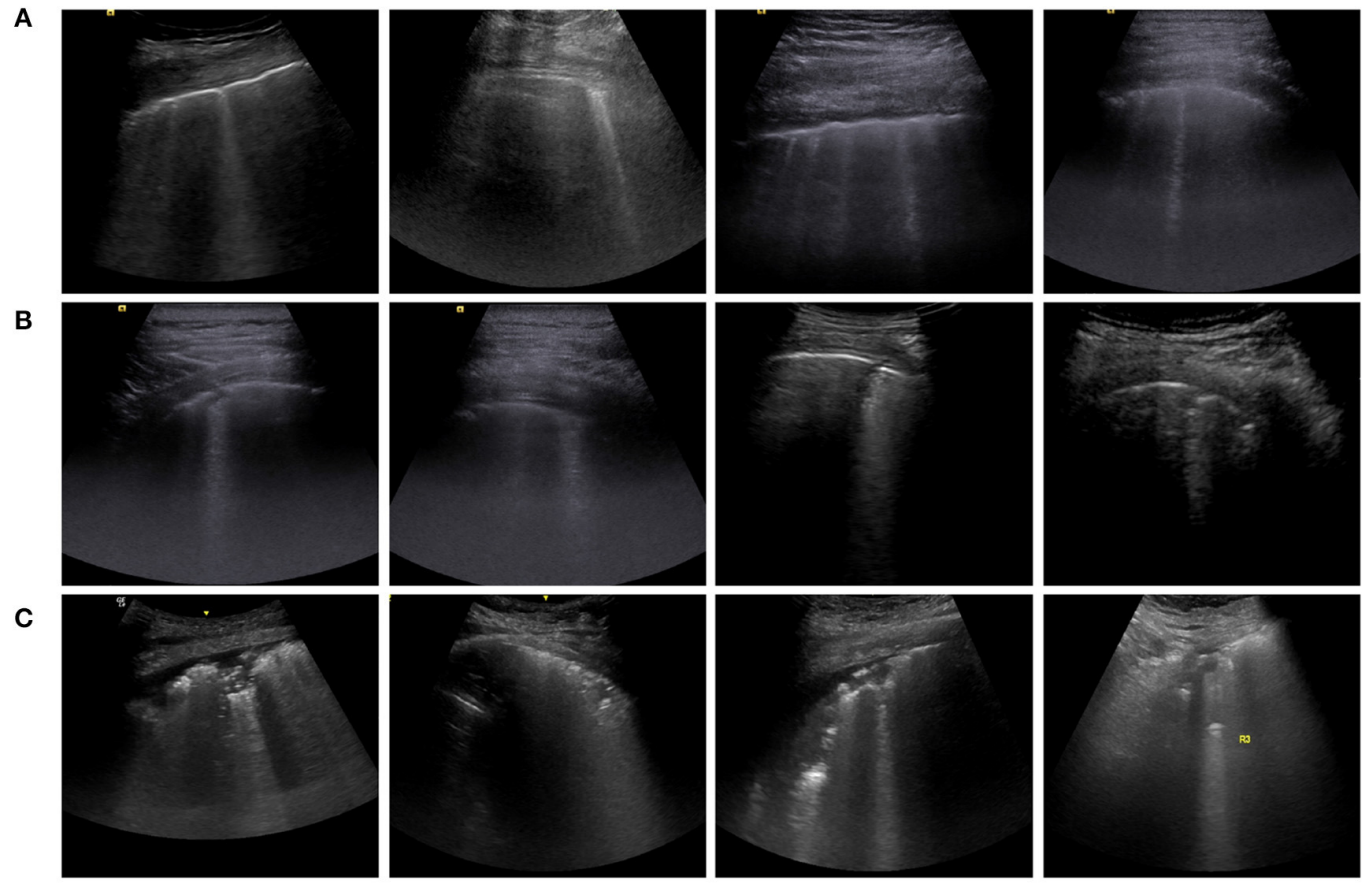
Typical examples of abnormal ultrasound cases related to coronavirus disease 2019 (COVID-19). **(A)** The pleural line is jagged or concave. **(B)** The pleural line is broken. **(C)** The scanning area shows a wide range of dense white areas, with or without large consolidation.

All lung ultrasound images were saved in .*jpg* format. The ultrasound equipment used was a Siemens ACUSON OXANA1 with 6C1HD and 9L4 probes. The probes were placed perpendicular to the chest wall and parallel to the frame on areas 1–6 on the left and right sides (for both the left and right sides: the upper and lower axillary areas, the front and side areas of the chest wall, and the area of breast attachment and of the shoulder blade angle on the posterior side). Notably, since patients in ICUs could not lie on their sides, only the anterior and lateral thorax (areas 1–4) were examined. According to a general ultrasound triage protocol (34), if an abnormal ultrasound sign was discovered (e.g., irregular pleural lines, B lines, consolidation, and pleural effusion), a static picture of the scan was saved. The examples of abnormal ultrasound cases are presented in Figure 1.

All biomedical indices were collected according to patients′ medical records, including Lymphocytes, C-reactive protein, Lactate dehydrogenase, Procalcitonin, and Interleukin 6. Table 1 shows that lymphocytes possess Q1 (1st Quartile) of 0.28 10^9^/L, Q3 (3rd Quartile) of 3.68 10^9^/L, and a median of 1.6 10^9^/L. C-reactive protein possesses Q1 of 0.5 mg/L, Q3 of 278.11 mg/L, and a median of 2.06 mg/L. Lactate dehydrogenase possesses Q1 of 116 IU/L, Q3 of 847 IU/L, and a median of 193 IU/L. Procalcitonin possesses Q1 of 0.01 ng/mL, Q3 of 9.25 ng/mL, and a median of 0.04 ng/mL. Interleukin 6 possesses Q1 of 1.5 pg/mL, Q3 of 1716 pg/mL, and a median of 1.71 pg/mL. Given that one patient usually has one ultrasonic examination and multiple pathological examinations, to make our work more reliable, biochemical indices from the pathological examination closest to ultrasonic examination were collected. Utilizing the summarized information, four experienced doctors made clinical diagnoses and annotated the corresponding data.

### 2.2. Overall Architecture

**Figure 2:**
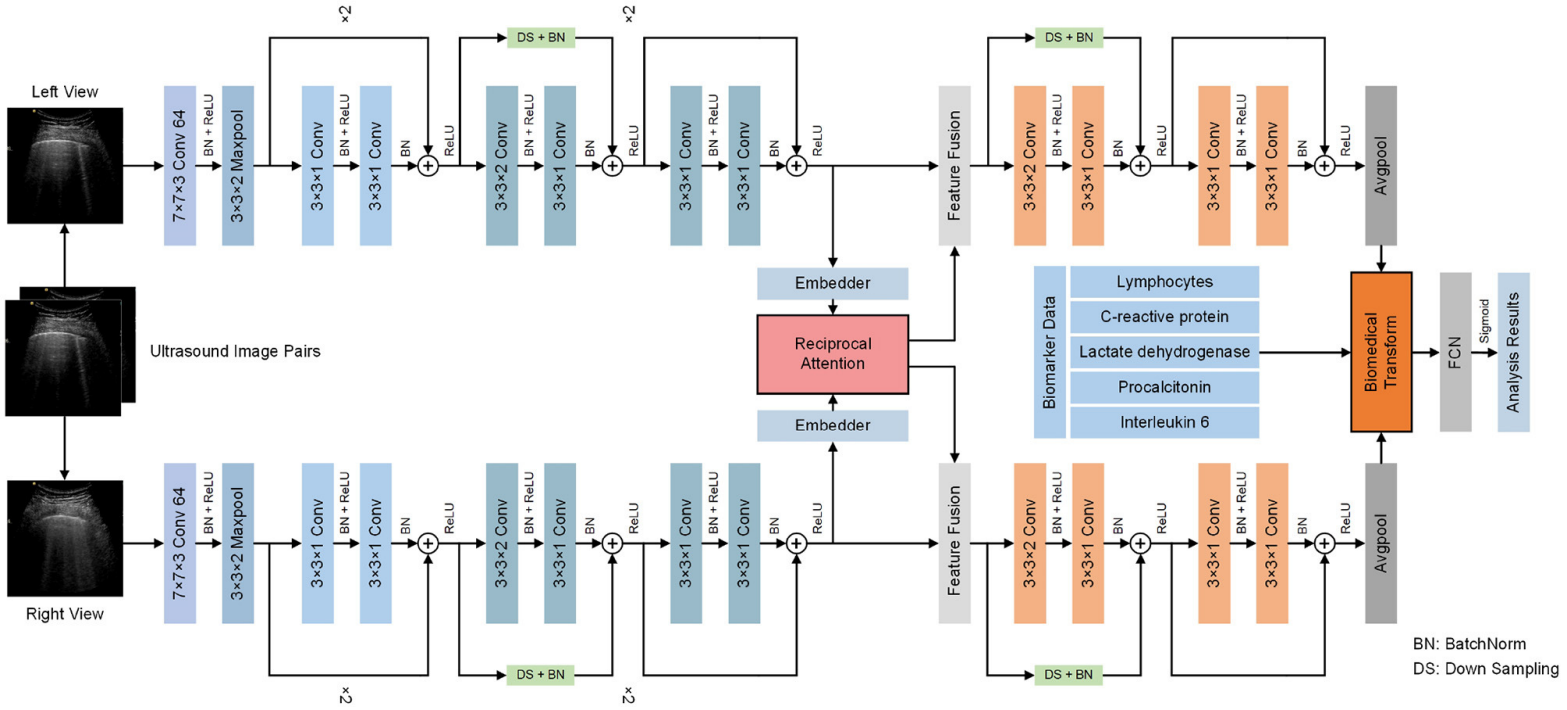
Overall flowchart of the proposed model. The proposed model receives multi-view ultrasound image pairs and biomedical indices of COVID-19 to conduct severity assessment tasks. The proposed reciprocal attention module tackles the multi-view ultrasound data and the proposed biomedical transform module tackles the biomedical data.

The architecture of the proposed model is presented in Figure 2. Generally, the proposed model receives multi-modal information of COVID-19 patients, containing ultrasound image pairs of two views and biomedical indices. Afterward, two branches of features are extracted from ultrasound image pairs. The two sets of features then undergo the proposed reciprocal attention module to acquire their attention features. Subsequently, attention features are further processed to obtain high-dimensional features. After average pooling, the high-dimensional features undergo the proposed biomedical transform module, where biomedical indices of corresponding patients are integrated to generate hybrid features. Exploiting hybrid features, our model conducts the final decision. Detailed architectures of reciprocal attention module and biomedical transform module are discussed in the following sections.

### 2.3. Reciprocal Attention Module

Different views of lung ultrasound images may contain complementary information. Hence, it is feasible to explore their inherent connections. Based on this, we propose a reciprocal attention module to acquire the attention feature from source view to target view bidirectionally, i.e., from left view to right view and from right view to left view simultaneously.

**Figure 3:**
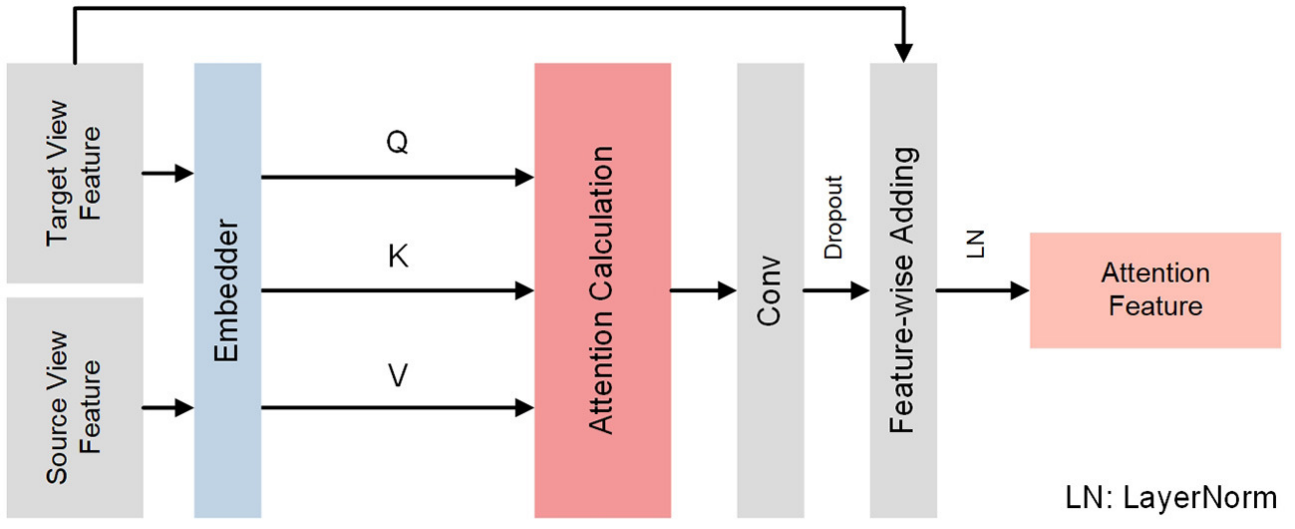
Detailed architecture of reciprocal attention module. Reciprocal attention module receives ultrasound image pairs and generates bidirectional attention features utilizing attention mechanism (33).

The detailed architecture of the reciprocal attention module is presented in Figure 3. The module receives features of two views extracted from previous CNN networks and calculates attention features utilizing attention mechanism (33). Initially, features of two views $\left\{{\text{F}}_{T},{\text{F}}_{S}\right\}\in {\mathbb{R}}^{H\times W\times C}$ are processed by an embedder to produce two independent embeddings $\left\{{\text{E}}_{T},{\text{E}}_{S}\right\}\in {\mathbb{R}}^{HW\times D}$, where *S* and *T* stand for source view and target view; *H*, *W* and *C* are the channel, height, and width of features; *D* is the dimension of embeddings. Then, according to (33), a query matrix **Q** ∈ ℝ^*HW*×*D*^, a key matrix **K** ∈ ℝ^*HW*×*D*^, and a value matrix **V** ∈ ℝ^*HW*×*D*^ are obtained as following:


(1) $$\begin{array}{l} \{\begin{array}{c} \text{Q}={\text{E}}_{T}{\text{W}}_{\text{Q}}\\ \text{K}={\text{E}}_{S}{\text{W}}_{\text{K}}\\ \text{V}={\text{E}}_{S}{\text{W}}_{\text{V}} \end{array} \end{array}$$

where {**W**_**Q**_, **W**_**K**_, **W**_**V**_} are learnable transform weights of query, key, and value, respectively. Exploiting acquired **Q**, **K**, **V**, reciprocal attention **RA**(·) ∈ ℝ^*HW*×*D*^ is calculated as follows:


(2) $$\begin{array}{l} \text{RA}\left(\text{Q},\text{K},\text{V}\right)=softmax\left(\frac{\text{Q}{\text{K}}^{T}}{\sqrt{D}}\right)\text{V} \end{array}$$

Similar to self-attention, reciprocal attention represents the weights from source embedding toward each element in target embedding. To conduct effective fusion with features of target view, reciprocal attention is firstly reshaped *via* convolution layers and then added to target features. Formally, attention feature is computed as follows:


(3) $$\begin{array}{l} {\text{F}}_{A}=\sigma \left({\text{F}}_{T}+\tau \left(\varepsilon \left(\text{RA}\right)\right)\right) \end{array}$$

where **F**_*A*_ is the attention feature after fusion, ε stands for reshaping convolution, τ is the dropout operation, and σ denotes the layer normalization. During the inference procedure, each view serves both as target and source and the other view serves as the opposite, yielding two branches of attention features corresponding to multi-view networks.

### 2.4. Biomedical Transform Module

Ultrasound images can provide partial information about the pulmonary lesions caused by COVID-19. However, COVID-19 causes wider damages to various organs and tissues, resulting in abnormal biomedical indices. Note that image features after CNNs are usually high-dimensioned while biomedical indices are low-dimensioned. Hence, it's significant to deploy biomedical indices and incorporate them with ultrasonic features. On the basis of this, we propose a biomedical transform module to undergo legitimate fusions between extracted graphic features and biomedical indices.

**Figure 4:**
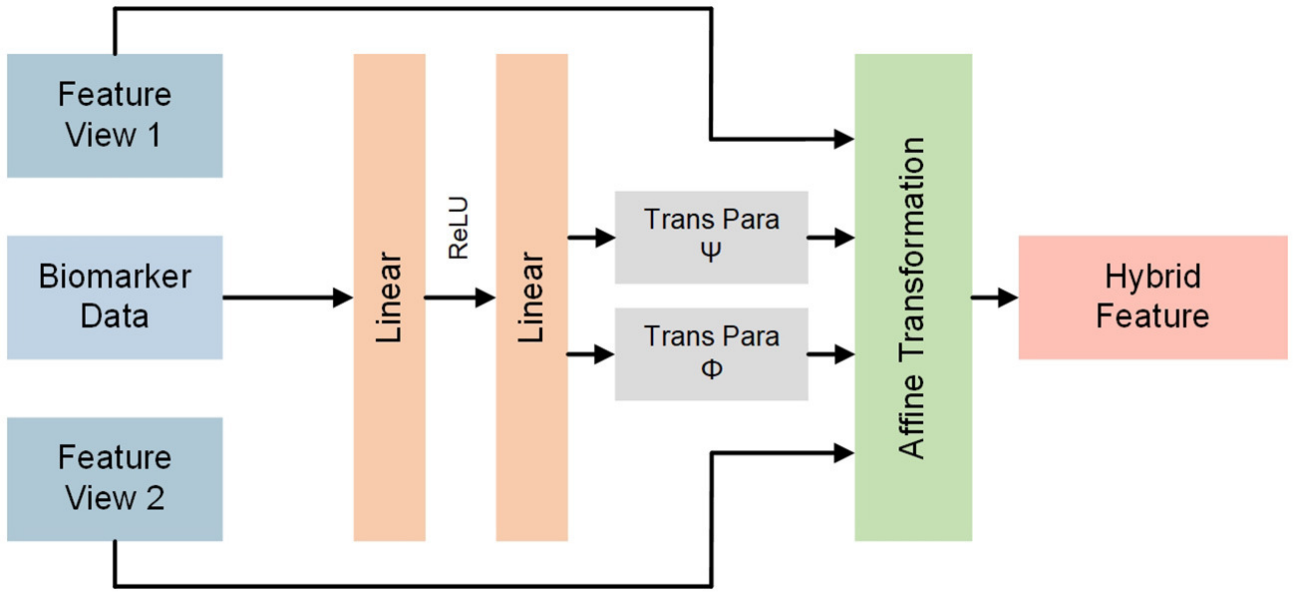
Detailed architectures of biomedical transform module. The biomedical transform module receives biomedical indices and generates parameters of affine transformation for ultrasonic features to obtain hybrid features.

The detailed architecture of biomedical transform module is illustrated in Figure 4. For any feature maps **F** ∈ ℝ^*H*×*W*×*C*^ in the network, **X** ∈ ℝ^*B*^ denotes the corresponding biomedical indices collected from the same patient and *B* is the dimension of biomedical indices. Let *F*_*i*_, *i* ∈ {1, 2, ⋯ , *C*} be the channel-wise features of **F**. Namely, [*F*_1_, *F*_2_, ⋯ , *F*_*C*_] = **F** where [·] denotes the concatenation operator. We aim to combine **X** and **F** through affine transformation. Concretely, an auxiliary network is built to generate transform parameters Ψ ∈ ℝ^*C*^ and Φ ∈ ℝ^*C*^, which can be indicated as follows:


(4) $$\begin{array}{l} \left\{\Phi ,\text{Ψ}\right\}=f_{an}\left(\text{X}\right) \end{array}$$

where *f*_*an*_ denotes the auxiliary network. Specifically, the auxiliary network is composed of multiple linear layers with ReLU functions to project **X** to Φ and Ψ. The auxiliary is jointly learned during the training procedure. Subsequently, affine transforms with the two learned parameters are applied to feature maps from corresponding patients to acquire hybrid features ${\text{F}}_{h}\in {\mathbb{R}}^{H\times W\times C}$. Formally, **F**_*h*_ is obtained as follows:


(5) $$\begin{array}{l} {\text{F}}_{h}=\left[\begin{array}{c} F_{h,1}\\ F_{h,1}\\ \cdots \\ F_{h,C} \end{array}\right]=\left[\begin{array}{c} \psi_{1}*F_{1}+\phi_{1}\\ \psi_{2}*F_{2}+\phi_{2}\\ \cdots \\ \psi_{C}*F_{C}+\phi_{C} \end{array}\right]=\text{Ψ}*\text{F}+\Phi \end{array}$$

where *F*_*h, i*_, *i* ∈ {1, 2, ⋯ , *C*} is the channel-wise features of **F**_*h*_, * denotes scalar multiplication, and +denotes scalar addition.

### 2.5. Evaluation

First, for severity assessment of COVID-19 patients, we define *Positive* for severe cases and *Negative* for mild cases. Then, *TP, TN, FP, FN* denote true positive, true negative, false positive, and false negative, respectively. To evaluate the performance of our proposed model, the following evaluating metrics are selected.

Accuracy: The most primitive evaluating metric in classification problems, defined as the percentage of correctly predicted results in the total sample. Formally, accuracy is defined as follows:


(6) $$\begin{array}{l} \text{Accuracy}=\frac{\text{TP}+\text{TN}}{\text{TP}+\text{FP}+\text{FN}+\text{TN}} \end{array}$$

Precision: It is defined as the proportion of correctly classified positive samples among all samples predicted to be positive and is a measure of how well the model can predict correct results among all predicted positive samples. Formally, precision is defined as follows:


(7) $$\begin{array}{l} \text{Precision}=\frac{\text{TP}}{\text{TP}+\text{FP}} \end{array}$$

Recall: The probability of predicting a correctly classified positive sample among all actually positive samples. Formally, recall is defined as follows:


(8) $$\begin{array}{l} \text{Recall}=\frac{\text{TP}}{\text{TP}+\text{FN}} \end{array}$$

F-score: The comprehensive measure of model precision and recall. We select two types of F-scores, F1-score and F1.5 score. F1-score treats precision and recall of equal significance and it is defined as follows:


(9) $$\begin{array}{l} \text{F}1\text{-score}=\frac{2\times \text{Recall}\times \text{Precision}}{\text{Recall}+\text{Precision}} \end{array}$$

F1.5-score emphasizes more on recall than precision, which is more rational in clinical practice (will be discussed in the following section). Formally, F1.5-score is defined as follows:


(10) $$\begin{array}{l} \text{F}1.5\text{-score}=\frac{13\times \text{Recall}\times \text{Precision}}{9\times \text{Recall}+4\times \text{Precision}} \end{array}$$

## 3. Experimental Results and Discussion

### 3.1. Implementation Details

To construct rational dataset for our multi-view multi-modal model, the aforementioned lung ultrasound images from 164 patients were separated into image pairs containing opposite views. Namely, ultrasound images of L1 were combined with those of R1 and the same for the remaining views. Under this strategy, one patient could produce multiple cases for training and test. Whereafter, biomedical indices were first normalized and then added into corresponding cases to form the complete dataset. In total, 834 cases were constructed, where 171 were severe and 663 were mild. Afterward, a set of 627 cases (498 mild and 129 severe) was selected as the training set, and the rest were selected as the test set (165 mild and 42 severe).

To reduce the influence of unusual data distributions on ultrasound images and improve the training efficiency of our model, original images were first normalized and then resized to 448 × 448 *pixels*. Common data augmentations like random flip and crop were applied to ultrasound images before entering the network.

The proposed network was implemented in Pytorch (35). The network was trained with a batch size of 32 and the total training epoch was set to 100. The initial learning rate was 1 × 10^−4^ and was adjusted according to ReduceLROnPlateau strategy (35). Specifically, the learning rate was reduced by a factor of 0.5 when the loss did not decline after 8 continuous epochs. Adam (36) with default parameters was adopted as the optimizer. Given that the number of mild cases and severe cases was disproportionate, Focal loss (37) was selected as the loss function to cope with imbalanced cases. Focal loss is defined as follows:


(11) $$\begin{array}{l} L_{FL}=\{\begin{array}{cc} -\alpha {\left(1-ȳ\right)}^{\gamma }logȳ, & y=1\\ -\left(1-\alpha \right){ȳ}^{\gamma }log\left(1-ȳ\right), & y=0 \end{array} \end{array}$$

where ȳ is the output of networks, *y* is ground truth, α and γ are parameters constructed to alleviate the negative influence brought by imbalanced data. During the training procedure, we set α = 0.25 and γ = 3.

### 3.2. Results of Severity Assessment of COVID-19 Patients

In this section, we present the experimental results of severity assessment in COVID-19 patients. To verify the advancement of our model, the performance of our model is compared to several other mainstream methods, namely, VGG (38), ResNet (39), DenseNet (40), SENet (41), SEResNet (41), Xception (42), InceptionV4 (43), and Sharifrazi et al. (26). We trained and tested the comparative methods with the same strategy and hyperparameters as the proposed model.

Table 2 summarizes the comparison of assessment results of COVID-19 patients. Our proposed model was referred to as RAB in this section. The experimental results indicated that the performance of the proposed RAB exceeds other models in almost all measurements. As for accuracy, our model achieved 92.75%, outperforming all other models. This indicates that RAB performs efficiently in recognizing both mild and severe cases. In terms of precision, RAB achieved 82.93%, inferior to several models. However, in early screening of COVID-19 patients, it counts more to recognize severe cases as many as possible, and it is tolerable to diagnose a tiny proportion of mild cases as severe ones but disastrous in turn. Therefore, precision is of less significance and emphasis should be placed more on recall. For recall, RAB yielded 80.95%, outperforming all other models. High performance in recall indicates that RAB performs preeminently in distinguishing severe cases from general COVID-19 cases, which is crucial clinically. Besides, for both F1 and F1.5 scores, our model also yielded the best outcomes, demonstrating that the comprehensive performance of our model overmatches all state-of-the-art models.

**Table 2:** Results of severity assessment in COVID-19 patients in terms of Accuracy (Acc), Precision (Pre), recall, and F-score.

**Model**	**Acc%**	**Pre%**	**Recall%**	**F1-score%**	**F1.5-score%**
VGG11BN (38)	91.30	90.00	64.29	75.00	70.48
DenseNet121 (40)	89.86	81.82	64.29	72.00	68.82
ResNet18 (39)	90.34	80.56	69.04	74.36	72.22
SENet (41)	90.82	92.59	59.52	72.46	66.87
SEResNet (41)	91.79	87.88	69.05	77.33	73.92
Xception (42)	90.34	77.50	73.81	75.61	74.91
InceptionV4 (43)	91.30	85.29	69.05	76.32	73.35
Sharifrazi et al. (26)	91.30	90.00	69.05	75.00	70.48
**RAB (Ours)**	**92.75**	**82.92**	**80.95**	**64.29**	**81.55**

**Figure 5:**
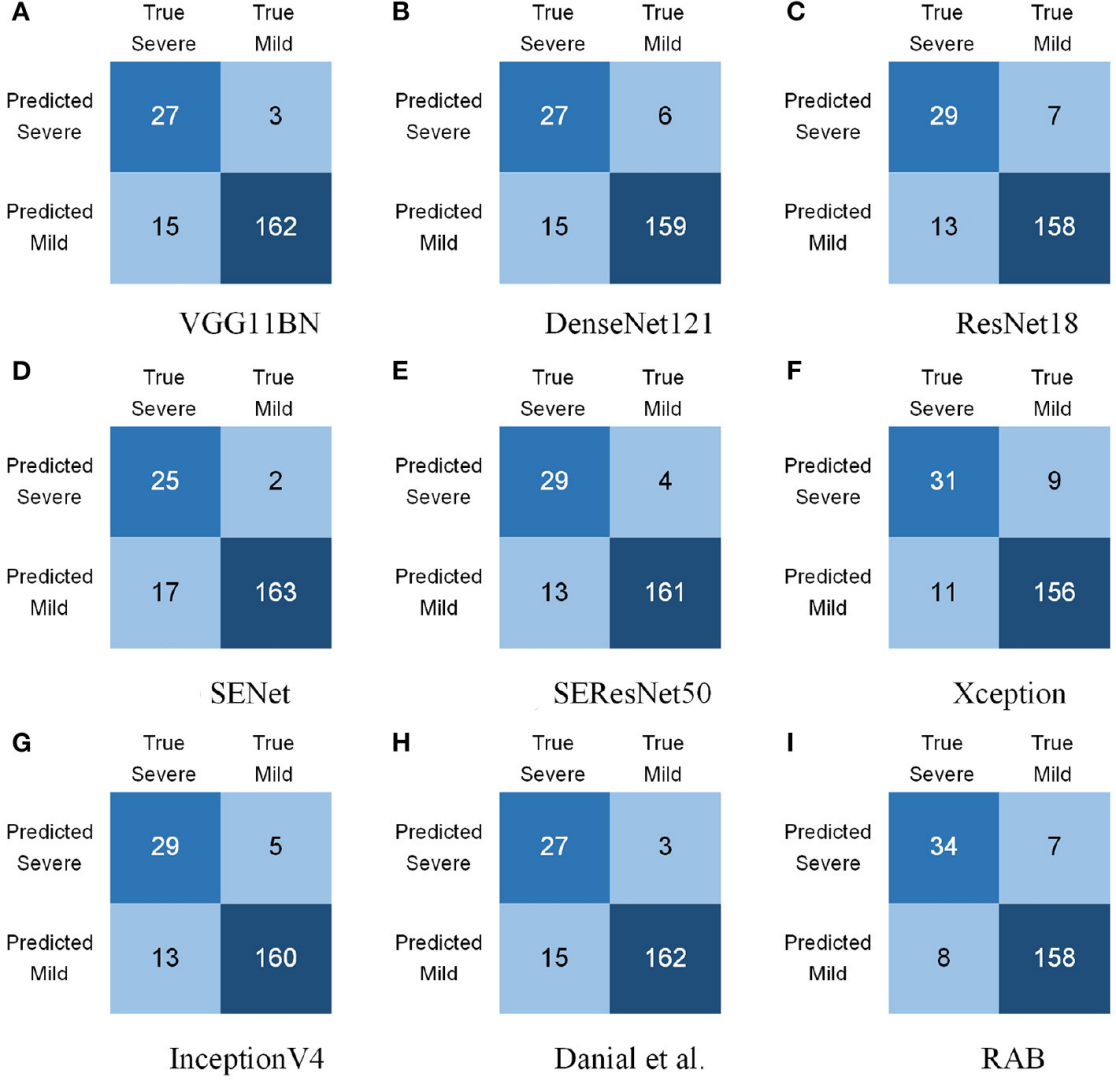
Confuse matrices of severity assessment for COVID-19 patients. **(A)** VGG11BN (38), **(B)** DenseNet121 (40), **(C)** ResNet18 (39), **(D)** SENet (41), **(E)** SEResNet (41), **(F)** Xception (42), **(G)** InceptionV4 (43), **(H)** Sharifrazi et al. (26) and, **(I)** RAB (Ours).

To make the experimental results more intuitive, the confusion matrices of all models were calculated and are presented in Figure 5. It is apparent to find that comparative methods tended to leave out more severe cases. For example, VGG11BN (38) and DenseNet121 (40) failed to recognize 15 cases out of 42 severe cases (Figures 5A,B), ResNet18 (39), SEResNet (41), and InceptionV4 (43) failed to recognize 13 severe cases (Figures 5C,E,G), and SENet (41) failed to recognize 17 severe cases (Figure 5D). The poor ability to identify severe COVID-19 cases restrains their applications. While RAB yielded the first place in recognizing severe cases and merely misdeemed 8 cases. Besides, it is noteworthy that Sharifrazi et al. (26) achieved plain results in recognizing COVID-19 cases because their method is aiming at X-ray images. X-ray images contain distinct boundaries for tissues and organs while ultrasound images usually have blurry ones. Hence, edge detection tends to produce mediocre outcomes for ultrasound images. We owe the remarkable performance of RAB to the proposed reciprocal attention module and biomedical transform module. The advancement of the two modules is discussed in the following section.

### 3.3. Ablation Studies

**Figure 6:**
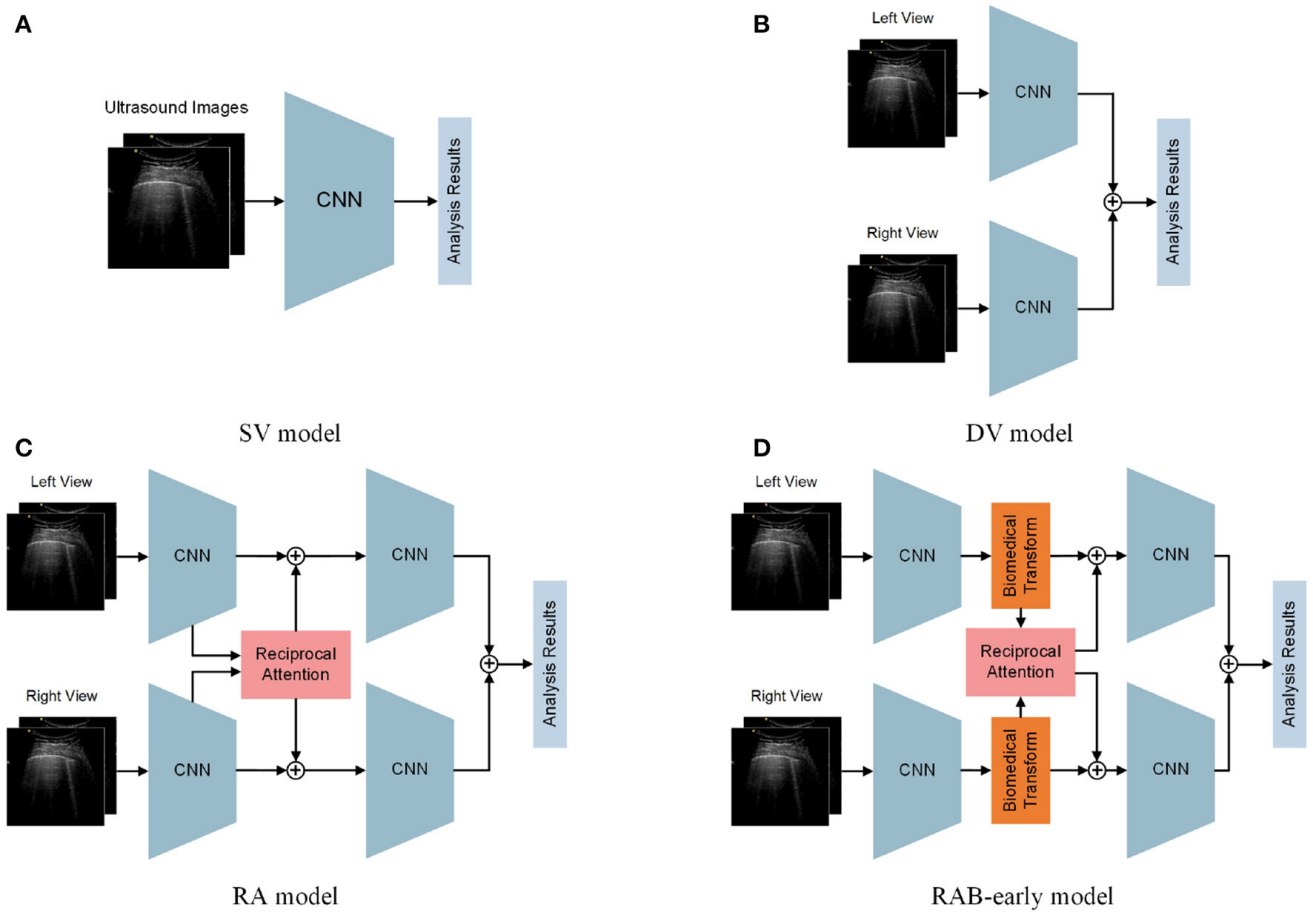
Structures of baseline models. **(A)** Single view (SV) Model. **(B)** Dual view model without reciprocal attention module and biomedical transform module (DV) Model. **(C)** Reciprocal attention model without biomedical transform module (RA) Model. **(D)** Biomedical transform module before reciprocal attention module (named RAB-early)-early Model.

In this section, to verify the advancement of the proposed reciprocal attention module and biomedical transform module, ablation experiments were conducted. The performance of our model is compared to several baseline methods. Except for our model, we also constructed 3 baseline models and 1 variant model. As shown in Figure 6, the 3 baseline models were as follows: Single View model (SV), Dual View model without reciprocal attention module and biomedical transform module (DV), and reciprocal attention model without biomedical transform module (RA). The 1 variant model is inserting the biomedical transform module before the reciprocal attention module (named RAB-early). Our proposed model is referred as RAB-late in this section. Similarly, we trained and tested the baseline methods with the same strategy and hyperparameters as RAB-late′s.

Table 3 summarizes the comparison of ablation results of COVID-19 patients. The experimental results indicated that the performance of the RAB-late model exceeds other baseline models in most measurements. As for accuracy, the RAB-late model achieved 92.75%, outperforming SV, DV, and RAB-early, the same as RA. In terms of precision, the RAB-late model achieved 82.93%, outperforming SV and RAB-early while inferior to DV and RA. The same as mentioned before, we focus more on recall. For recall, the RAB-late model yielded 80.95%, overmatching most other models. Note that the RAB-early model achieved the best recall of 85.71% while it had only 59.01% of precision. Such a model is fallacious in clinical use. For both F1 and F1.5 scores, the RAB-late model yielded the best outcomes.

**Table 3:** Results of ablation experiments in terms of Acc, Pre, recall, and F-score.

**Model**	**Acc%**	**Pre%**	**Recall%**	**F1-score%**	**F1.5-score%**
SV	90.34	80.56	69.04	74.36	72.22
DV	91.79	83.78	73.81	78.48	76.62
RA	92.75	88.57	73.81	80.52	77.80
RAB-early	85.02	59.02	85.71	69.90	75.24
**RAB-late**	**92.75**	**82.92**	**80.95**	**81.93**	**81.55**

**Figure 7:**
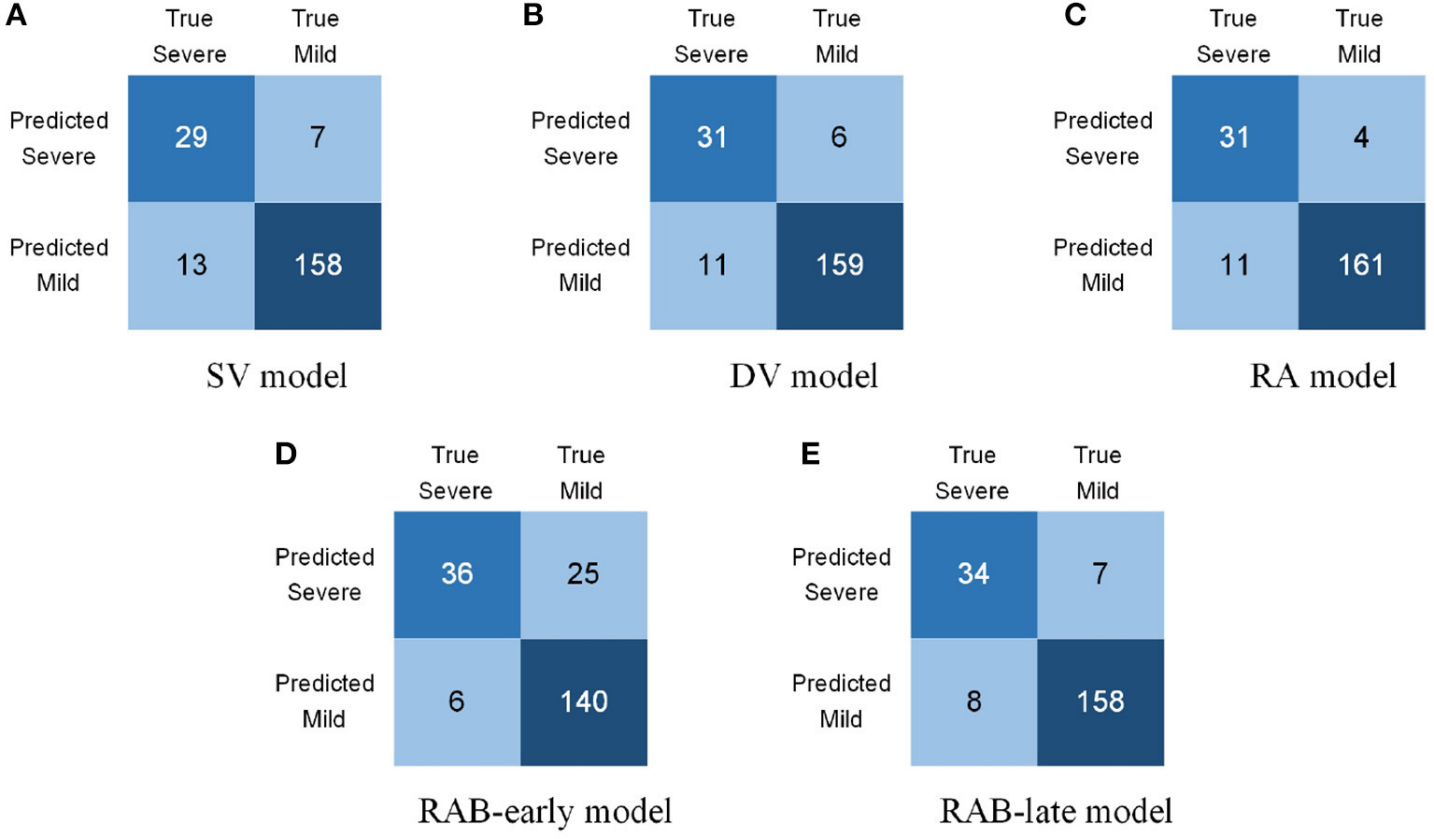
Confuse matrices of ablation experiments. **(A)** SV Model, **(B)** DV Model, **(C)** RA Model, **(D)** RAB-early Model, and **(E)** RAB-late Model.

Additionally, the confusion matrices of all 5 models are shown in Figure 7. As shown in Figure 7A, among 42 severe cases, the SV model failed to recognize 13 cases, leading to a disappointing recall. DV model surpassed the SV model in both recall and precision, demonstrating the validity of a dual-view strategy (Figure 7B). Moreover, the RA model strengthened the model's ability in identifying mild cases and improving model's precision, confirming the advancement of our proposed reciprocal attention module (Figure 7C). Notably, as presented in Figure 7D, the RAB-early model identified 36 severe cases out of the total 42 testing cases, yielding the best performance in recall, whereas it failed to classify 25 mild cases, far inferior to other models. In contrast, the RAB-late model maintained the most remarkable in both precision and recall and subsequent F-scores. The reason account for such gaps lies in the intrinsic structures of RAB-early and RAB-late, namely, the sequence of reciprocal attention and biomedical transform. According to aforementioned methodology, the reciprocal attention module seeks for connections of multiple views on the hypothesis that different views may possess complementary information. Nevertheless, such mechanism is futile when confronting identical features. In the RAB-early model, before the reciprocal attention module, biomedical transform is conducted to dual-view features with parameters generated from the same biomarker data, bringing identical factors to subsequent blocks and those identical factors restrain reciprocal attention mechanism. Whereas, the DAVB-late model encounters no similar restraints.

**Figure 8:**
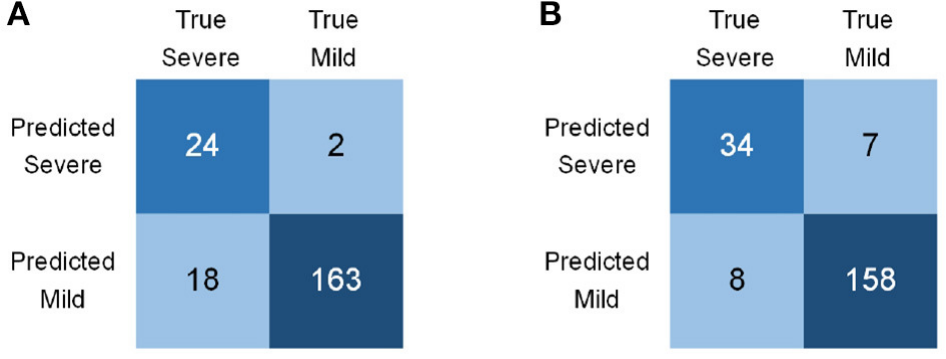
Confuse Matrices of different loss functions. **(A)** BCE Loss model. **(B)** Focal Loss model.

Apart from the two proposed modules, the adopted focal loss function is also discussed. We replaced the focal loss with a more general loss function, BCE Loss, to train and test our model. Table 4 summarizes the comparison of different loss functions. The result indicates that the focal loss apparently elevated our model's overall performance. Note that the BCE Loss model generated extremely uneven precision and recall. The confusion matrices are shown in Figure 8. BCE Loss model failed to recognize 18 severe cases out of 42, distinctly inferior to the focal loss model. Technically, focal loss reduces the weights of categories with vast majority, thus applicable for the imbalanced COVID-19 data.

**Table 4:** Results of different loss functions in terms of Acc, Pre, recall, and F-score.

**Loss function**	**Acc%**	**Pre%**	**Recall%**	**F1-score%**	**F1.5-score%**
$L_{BCE}$	90.34	92.31	57.14	70.59	64.73
$L_{FL}$	**92.75**	**82.92**	**80.95**	**81.93**	**81.55**

### 3.4. Visualization

**Figure 9:**
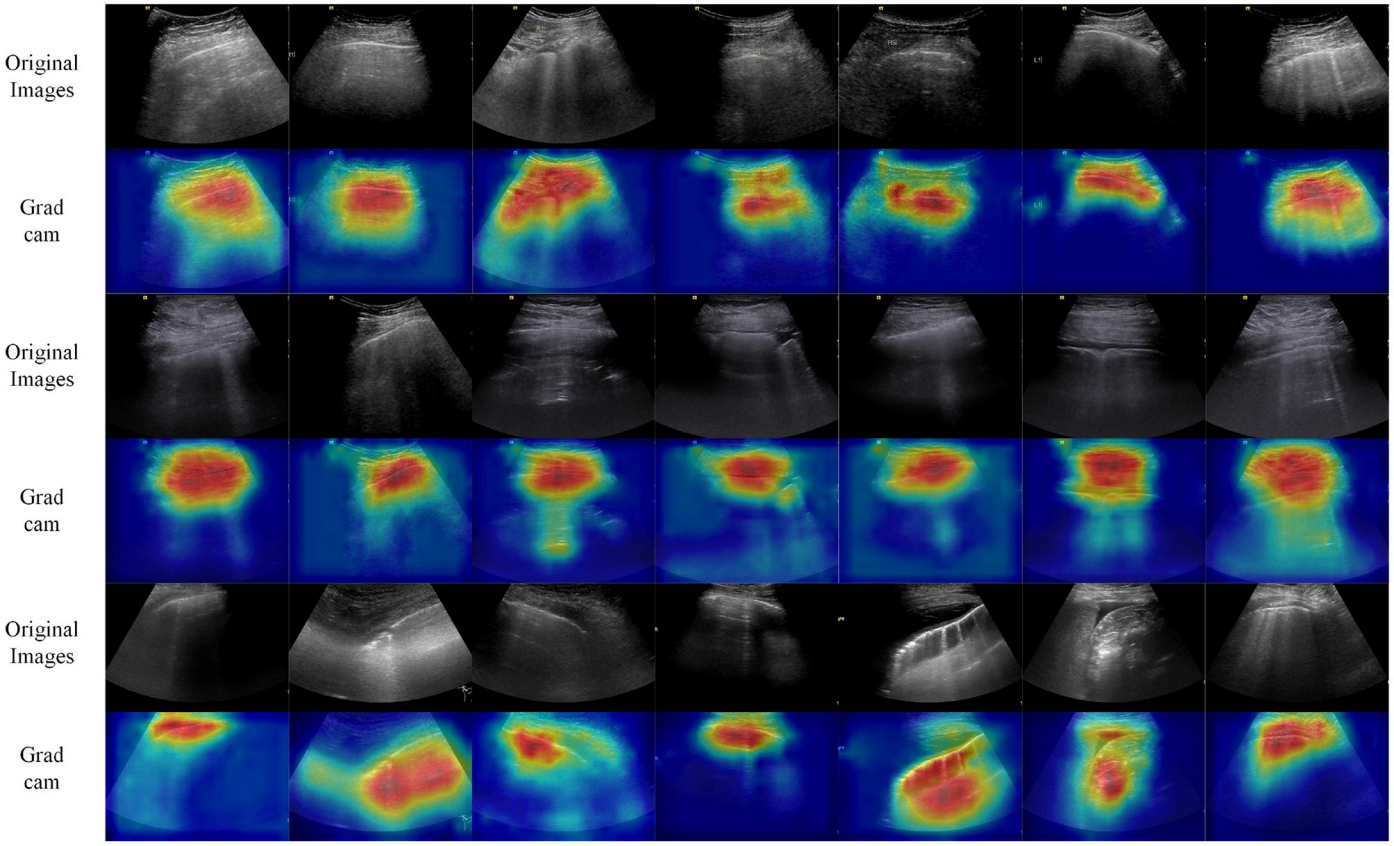
Nidus-related visualization of ultrasound images. Using grad-CAM (44), the proposed model could highlight the image areas that are most relevant to COVID-19.

To interpret that our model has indeed learned certain abnormal signs to undergo an assessment of COVID-19 patients, grad class activation map (grad-CAM) (44) was used to visualize the most disease-indicative image areas learned by our model. Figure 9 presents the heat maps relevant to COVID-19 generated by the grad-CAM of our model. Distinctly, it demonstrates in Figure 9 that our model has detected several abnormal signs like irregular pleural lines, B lines, consolidation, and pleural effusion as standards to conduct downstream tasks.

**Figure 10:**
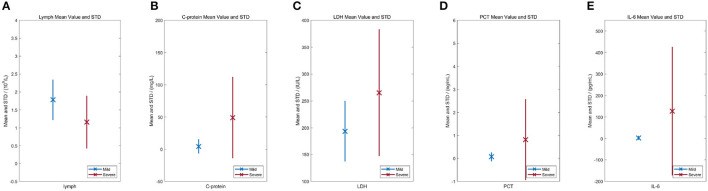
Statistical characteristics of biomedical indices. × stands for the mean value. The red and blue bar stands for the standard deviation for severe and mild cases, respectively. **(A)** Lymphocyte (absolute value). **(B)** C-reactive protein. **(C)** Lactate dehydrogenase. **(D)** Procalcitonin. **(E)** Interleukin-6.

Additionally, to further interpret the advancement of biomedical indices in an assessment task, the statistical characteristics of biomedical indices are also considered. As shown in Figure 10, the mean value and SD of those data are demonstrated with × and bars with different colors indicate mild and severe cases. In Figure 10, the red and blue bars represent the SD for all biomedical indices, and the length of each bar represents the data range from *mean* − *std* to *mean* + *std*, standing for the approximate distribution of biomedical indices for mild and severe cases. For C-reactive protein, lactate dehydrogenase, procalcitonin, and interleukin 6, the data distributions of mild and severe patients overlap completely. For absolute lymphocyte value, there also exists overlaps. Data distributions of biomedical indices indicate that mild and severe cases possess certain but no sheer disparity, which brings challenges for manual judgments. Whereas, our model successfully exploits the meritorious information in biomedical indices and incorporates it into multi-modal data.

Still, our model owns several flaws. Subject to insufficient laboratory data, the scale of our dataset is not as abundant as million-level ones, imposing restrictions on our model. In addition, for the same reason, not all patients have complete biochemical indices available and we simply replaced those void data with average ones. Those missing data may lead to restraints to on our model as well. Experiments with data of larger-scale and better integrity will be carried out in future work.

## 4. Conclusion

In this paper, we present a novel automated multi-view multi-modal model to assess the severity of COVID-19 patients exploiting ultrasound images and biomedical indices. The model has a dual-view structure and receives ultrasound images along with biomedical indices to generate comprehensive features. Specifically, the proposed reciprocal attention module acquires inherent connections between multiple views. and the proposed biomedical transform module integrates biomedical indices with extracted ultrasonic features to form hybrid features.

We have evaluated our model on compound datasets composed of ultrasound images and biomedical indices of COVID-19 patients. Experimental results demonstrate that our method outperforms all other state-of-the-art methods with better comprehensive performance. Further ablation studies and discussions consistently substantiate the rationality and advancement of our model. In the future, the model will be extended to wider ranges of modalities and larger scales of data.

## Data Availability Statement

The raw data supporting the conclusions of this article will be made available by the authors, without undue reservation.

## Ethics Statement

The studies involving human participants were reviewed and approved by the Institutional Ethics Board of Zhongnan Hospital of Wuhan University (No. 2020042). The patients/participants provided their written informed consent to participate in this study.

## Author Contributions

YLi, HZ, LZ, CF, and MW: conceptualization. YLi, HZ, and TG: methodology and software. YLi, TG, YLiu, LZ, TX, and CF: validation. YLi, HZ, TG, and TX: formal analysis. YLi, HZ, TG, and YLiu: investigation and visualization. YLi, HZ, TG, YLiu, TX, XC, and MW: resources and data curation. YLi, TG, YLiu, LZ, TX, XC, CF, and MW: writing—original draft preparation. YLi, HZ, TG, YLiu, LZ, TX, XC, CF, and MW: writing—review and editing. HZ, LZ, CF, and MW: supervision and project administration. HZ and MW: funding acquisition. All authors have read and agreed to the published version of the manuscript.

## Funding

This study was funded by Hubei Provincial Natural Science Foundation (Grant No. 2020CFB729), Chinese Ultrasound Doctors Association, Science and Technology New Star Project (Grant No. KJXX2020002), Health Commission of Hubei Province Youth Talent Project (Grant No. WJ2021Q044), Zhongnan Hospital of Wuhan University Science, Technology and Innovation Seed Fund (Grant No. 2019090), research fund from the Medical Sci-Tech Innovation Platform of Zhongnan Hospital, Wuhan University (Grant No. PTXM2020027), Kuanren Talents Program of The Second Affiliated Hospital of Chongqing Medical University (Grant No. 2021240308), and National Natural Science Foundation Youth Project (Grant No. 81801714).

## Conflict of Interest

XC was employed by BGI Research. The remaining authors declare that the research was conducted in the absence of any commercial or financial relationships that could be construed as a potential conflict of interest.

## Publisher's Note

All claims expressed in this article are solely those of the authors and do not necessarily represent those of their affiliated organizations, or those of the publisher, the editors and the reviewers. Any product that may be evaluated in this article, or claim that may be made by its manufacturer, is not guaranteed or endorsed by the publisher.
